# Plasma metabolomic profiles as affected by diet and stress in Spanish goats

**DOI:** 10.1038/s41598-021-91893-x

**Published:** 2021-06-15

**Authors:** Phaneendra Batchu, Thomas H. Terrill, Brou Kouakou, Zaira M. Estrada-Reyes, Govind Kannan

**Affiliations:** Agricultural Research Station, Fort Valley State University, 1005 State University Drive, Fort Valley, GA 31030 USA

**Keywords:** Biochemistry, Physiology, Biomarkers

## Abstract

The effects of high-condensed tannin (CT) diet combined with preslaughter stress have not been studied at the metabolome level in goats. This study was conducted to determine the effects of feeding sericea lespedeza (SL; *Lespedeza cuneata*), a high-CT legume, and transportation stress on plasma metabolome in goats. Uncastrated male Spanish goats (age = 8 months; BW = 26.0 ± 0.48 kg) were either fed ground ‘Serala’ SL hay (SER), bermudagrass (*Cynodon dactylon*) hay (BG), or bermudagrass hay-dewormed goats (BG-DW; Control) at 75% of intake, with a corn-based supplementation (25%) for 8 weeks (n = 12/Diet). At the end of the trial, goats were subjected to one of two stress treatments (ST): transported for 90 min to impose stress (TS) or held in pens (NTS) before slaughtering, in two replicates. Live and carcass weights, and blood samples were collected at 0, 30, 60 and 90 min of transportation or holding time (Time). The data were analyzed using MIXED procedures in SAS and metabolomics data were analyzed using the R software. When measured after ST, SER group had the lowest body weight (*P* < 0.05) among the three diet groups. Carcass weights were high in the BG-DW, low in SER, and intermediate in BG group. Plasma creatine concentrations decreased over Time (*P* < 0.01) in the TS goats in all diet groups. Meat crude protein percentages were higher (*P* < 0.05) in SER (22.5 ± 0.22) and BG-DW (22.3 ± 0.22) groups compared to the BG group (21.6 ± 0.22). At the metabolome level, SER group had the lowest (*P* < 0.05) glycine, alanine, threonine, taurine, trans-hydroxyproline, methionine, and histidine concentrations and highest (*P* < 0.01) lysine and citrulline concentrations among the Diet groups. Butyric acid, concentration was higher (*P* < 0.05) in the SER group compared to BG group. Eight medium- and long-chained acylcarnitines were higher (*P* < 0.05) in the BG-DW group than SER or BG groups. In general, amino acid levels decreased and acylcarnitine increased with Time (*P* < 0.05) in all groups. Sericea diet can be beneficial in enhancing stress coping abilities in goats due to elevated butyrate, lysine, and citrulline levels; however, SER resulted in lower energy level in goats compared to BG or BG-DW groups. Fatty acid metabolism is the main energy pathway in all groups during prolonged stress. Inclusion of certain varieties of SL in the diet must be carefully controlled to prevent possible negative effect.

## Introduction

Goat production is a growing industry in the southeastern US because of availability of forages and growing demand for goat meat, particularly organically produced meat^1^. Controlling gastrointestinal parasites^2^ and preharvest stress^3^ are essential aspects of meat goat management in regions with high rain fall and humidity and without adequate number of goat processing facilities that necessitates extended live haul processes.

Previous studies have shown that transportation stress elicits metabolic and physiological changes^4^ that impact health and production variables, including immune response and body weight in livestock^5^. Overuse of anthelmintic drugs has increased incidence of anthelmintic resistance; thus, it is necessary to develop farm management systems that can maintain high levels of animal productivity with reduced reliance on anthelmintics^2^.

Feeding diets rich in condensed tannins (CT) has been used to alleviate the negative impact of transportation stress on carcass and meat quality^6^. In addition, the tannin-protein complexes that are formed when tannin-rich diets are fed to ruminants are reported to be harmful to gastrointestinal nematodes^7^. Sericea lespedeza (SL; *Lespedeza cuneata*) is a perennial, drought tolerant legume that grows well on acidic soils with low fertility^8^ and contains high concentration of CT. Sericea is known to have antioxidant properties that reduce free radical production^9^. In vitro studies using SL have revealed presence of natural antioxidant properties in this species^10^.

Metabolomics has emerged as a robust high-output analytical method for identifying metabolites in ruminants in response to stress and diet^11,12^. It is the large-scale study of low molecular weight molecules within cells, biofluids, tissues, or organisms, and these metabolites can be influenced by genetic and environmental factors^13^. Metabolomics can be used to identify the metabolite biomarkers and biological pathways of importance in both basic and applied research^14^. Typically, compounds are identified by comparing the measured masses to those of known metabolites stored in databases such as HMDB^15^, LipidMaps^16^ and Metlin^17^.

Studies on the effects of diet and stress on plasma metabolomics in goats are very limited. Although it has been reported that SL diet can have several positive effects in goats, to the best of our knowledge, there has been no study on the combined effects of SL and preslaughter stress at the metabolome level in goats. The overall aim of the present study was to determine the separate and combined effects of feeding a diet rich in CT and short-term simulated preslaughter stress on live and carcass weights, meat composition, and plasma metabolome in Spanish goats.

## Methods

### Animals

The animal care protocol for this research was reviewed and approved by the Fort Valley State University’s Agricultural and Laboratory Animal Care and Use Committee (ALACUC) prior to beginning of the experiment. In a Completely Randomized Design, 36 uncastrated male Spanish goats (body weight 26.1 ± 0.16 kg) were assigned to 3 treatments: ground ‘Serela’ SL hay (SER), bermudagrass hay (BG), or bermudagrass hay-dewormed goats (BG-DW; Control) at 75% of intake, with a corn-based supplementation (25%) for 8 weeks (n = 12 goats/group). Goats were kept in individual pens during the feeding trial and feed intake and average daily gain were monitored. At the end of the feeding period, all animals were weighed and transported to the processing facility and held overnight in holding pens prior to slaughter. On the day of slaughter, half the goats were transported (TS) for 90 min to impose stress (stress treatment, ST) and the remaining goats were held in pens (non-transported, NTS) before slaughtering. The trial was conducted on two different days (replicate) using 18 goats on each day. All goats were weighed prior to beginning of transportation and after transportation. Animals were slaughtered using humane procedures.

### Feed sampling

Samples of hay and concentrates collected at the beginning, middle, and end of the experiment were pooled and analyzed for nutrient content at a commercial laboratory (Dairy One Forage Laboratory, Ithaca, New York) and estimates of diet nutrient analyses were calculated. These values are presented along with the ingredients used to formulate concentrate portions in Table 1. The ‘Serala’ sericea lespedeza hay samples analyzed had a total CT content of 5.1% and the bermudagrass hay samples had virtually no CT content.

**Table 1:** Composition of hay, concentrate, and hay plus concentrate samples and the ingredients used to formulate the concentrate portions of BG and SER diets.

Item	Bermudagrass diet (BG)	‘Serala’ sericea lespedeza diet (SER)
**Composition**		
Hay		
Dry matter, %	90.8	91.6
Crude protein, %	14.7	15.3
Acid detergent fiber, %	33.5	58.9
Neutral detergent fiber, %	68.1	74.0
Total digestible nutrients, %	57.0	53.0
Concentrate		
Dry matter, %	86.8	88.2
Crude protein, %	29.1	31.2
Acid detergent fiber, %	9.1	9.1
Neutral detergent fiber, %	13.7	13.8
Total digestible nutrients, %	83.0	83.0
Hay + concentrate		
Crude protein, %	18.3	19.3
Acid detergent fiber, %	27.4	46.5
Neutral detergent fiber, %	54.5	58.9
Total digestible nutrients, %	63.5	60.5
**Ingredients**		
Hay + concentrate		
Bermudagrass hay, %	75.0	0
‘Serala’ sericea lespedeza hay, %	0	75.0
Ground corn, %	6.0	6.0
Soybean meal, %	12.0	2.7
Poultry fat, %	1.6	1.6
Molasses (dry), %	3.0	3.0
TM salt (red salt), %	0.7	0.5
Vitamin Premix, %	0.7	0.5
Calcium carbonate, %	1.0	0
Biofos (mono calcium phosphate), %	0	0.7
Concentrate alone		
Ground corn, %	24.0	24.0
Soybean meal, %	48.0	50.8
Poultry fat, %	6.4	6.4
Molasses (dry), %	12.0	12.0
TM salt (red salt), %	2.8	2.0
Vitamin premix, %	2.8	2.0
Calcium carbonate, %	4.0	0
Biofos (mono calcium phosphate), %	0	2.8

### Blood and meat sampling

Blood samples were collected every 30 min from individual animals by jugular venipuncture into the vacuum tubes containing K_2_EDTA and kept on ice until separation of plasma. Blood tubes were centrifuged at 3000 rpm for 10 min to separate the plasma. Plasma aliquots were stored at − 20 °C until analysis. Goats were slaughtered and the carcasses were weighed and stored at 2 °C. After 24 h of cooler storage, each carcass was then weighed again, and loin/rib chops were collected, vacuum packed, and stored at − 20 °C for proximate analysis.

### Proximate analysis

Percent dry matter, ash, fat, and protein were analyzed using AOAC^18^ procedures. Samples for proximate analysis were thawed at 4 °C. Total protein was determined using a carbon/nitrogen analyzer (Vario MAX cube, Elementar, NJ), standardized with l-glutamic acid. Approximately 0.5 g of meat samples were taken in metal crucibles and placed in the auto sampler. Percent nitrogen was determined by combustion (900 °C), and these values were multiplied by 6.25 to obtain protein percentages. For dry matter analysis, approximately 2 g samples were placed in crucibles and kept in an oven at 100 °C overnight. The crucibles were taken out, placed in a desiccator to cool, and then weighed to calculate the percent dry matter. The crucible with dried sample from the dry matter determination was placed in a furnace (Thermolyne, Type 48000 Furnace, Augusta, Georgia) at 600 °C for at least 2 h. The crucibles with ashed samples were taken out, placed in the desiccator to cool, and then weighed to calculate percent ash. Ether extract was determined using a Soxtec 8000 (Foss North America, Eden Prairie, MN) Extraction Unit.

### Metabolomics

An aliquot (200 µL) of plasma samples from SER, BG, and BG-DW groups collected at 0, 30, 60 and 90 min of transportation from TS and NTS animals were shipped on dry ice to the University of Alberta (Edmonton, AB Canada) for targeted metabolomics analysis. A total of 136 metabolites were analyzed using DI/LC–MS/MS TMIC PRIME Assay.

A targeted quantitative metabolomics approach was employed to analyze the samples using a combination of direct injection mass spectrometry with a reverse-phase LC–MS/MS custom assay. This custom assay, in combination with an ABSciex 4000 Qtrap (Applied Biosystems/MDS Sciex) mass spectrometer, can be used for the targeted identification and quantification of up to 150 different endogenous metabolites, including amino acids, acylcarnitines, biogenic amines and derivatives, uremic toxins, glycerophospholipids, sphingolipids, and sugars^19^. The method combines the derivatization and extraction of analytes, and the selective mass-spectrometric detection using multiple reaction monitoring (MRM) pairs. Isotope-labeled internal standards and other internal standards were used for metabolite quantification. The custom assay contains a 96 deep-well plate with a filter plate attached with sealing tape, and reagents and solvents used to prepare the plate assay. The first 14 wells were used for one blank, three zero samples, seven standards and three quality control samples. For all metabolites except organic acids, samples were thawed on ice and were vortexed and centrifuged at 13,000×*g*. Each sample (10 µL) was loaded onto the center of the filter on the upper 96-well plate and dried in a stream of nitrogen. Subsequently, phenyl-isothiocyanate was added for derivatization. After incubation, the filter spots were dried again using an evaporator. Extraction of the metabolites was then achieved by adding 300 µL of extraction solvent. The extracts were obtained by centrifugation into the lower 96-deep well plate, followed by a dilution step with MS running solvent.

For organic acid analysis, 150 µL of ice-cold methanol and 10 µL of isotope-labeled internal standard mixture was added to 50 µL of sample for overnight protein precipitation, followed by centrifugation at 13,000×*g* for 20 min. The supernatant (50 µL) was loaded into the center of wells of a 96-deep well plate, followed by the addition of 3-nitrophenylhydrazine (NPH) reagent. After incubation for 2 h, BHT stabilizer and water were added before LC–MS injection.

Mass spectrometric analysis was performed on an ABSciex 4000 Qtrap^®^ tandem mass spectrometry instrument (Applied Biosystems/MDS Analytical Technologies, Foster City, CA) equipped with an Agilent 1260 series UHPLC system (Agilent Technologies, Palo Alto, CA). The samples were delivered to the mass spectrometer by an LC method followed by a direct injection method. Data analysis was done using Analyst 1.6.2.

### Statistical analysis

The body weight, carcass weight, and meat proximate concentration data were analyzed using Mixed Procedures in SAS (release 9.1, SAS Institute, Cary, NC, USA) as a Completely Randomized Design with Diet and ST as fixed effects. When Diet, ST, or Diet × ST effects were significant by ANOVA at *P* < 0.05, the means were separated using the LSD test.

Initial analysis of metabolomics data was performed using omu package in R software. This package assigns hierarchical metadata from the Kyoto Encyclopedia of Genes and Genomes (KEGG)^20^ to each metabolite in order to create intuitive figures for visualizing the data. Principal Component Analysis (PCA), an unsupervised clustering technique, was used to reduce dimensionality of data and examine the intrinsic variation in the data set. To analyze selected metabolite groups of interest (amino acids/organic acids, and acyl carnitines), a classical analytical approach to assess group-wise differences in a univariate parameter-by-parameter fashion (*t* test) was used. The Student’s *t* test (α = 0.05) was utilized to analyze each metabolite between two experimental groups: BG vs SER and BG-DW vs SER. Univariate volcano plots were generated by plotting the fold change against false discovery rate adjusted *P*-value. Volcano plots show data based on their *t* test *P*-values vs. their fold change and are generally used to rapidly identify and visually display significant differences in metabolites.

To perform a more robust analysis, metabolomics data within TS and NTS groups were analyzed using the R software using a Repeated Measures Model. Fixed effects included diet and time, and animal was added as random effect. Interaction effects were also included in the model. To evaluate changes in plasma metabolome caused by Diet (SER vs. BG) effect with an ST group, a hierarchical clustering analysis using the degree of abundance was conducted in which metabolites with identical abundance patterns were clustered together. Clustering was visualized by means of heatmap dendograms. Proc Mixed in SAS provides a very flexible environment to model many types of repeated measures data, including repeated in time. The metabolites that were found significant were further analyzed using Mixed procedure in SAS to compare the main effects of Diet (SER, BG, BG-DW), Time (0, 30, 60, and 90 min), and their interaction, and the means were separated using the LSD test when significant by ANOVA at *P* < 0.05.

### Ethics approval and consent to participate

All methods in this study were carried out in accordance with relevant guidelines and regulations. The animal care protocols were approved by the FVSU Agricultural and Laboratory Animal Care and Use Committee prior to beginning of the study following the ADSA-ASAS-PSA Guide for Care and Use of Agricultural Animals in Research and Teaching.

## Results

### Feed intake and average daily gain

Diet did not influence feed intake; however, average daily gain was affected by Diet (*P* < 0.01). The average daily gains were 57.0 ± 4.72, 94.1 ± 5.12, and 127.3 ± 4.94 g, respectively, in SER, BG, and BG-DW groups.

### Live and carcass weights and carcass yield

Diet main effect was significant for the goats’ live weight when measured after ST (*P* < 0.05; Table 2) with SER group having the lowest body weight among the three dietary treatment groups. Interaction means revealed that the goats subjected to BG-DW plus NTS had the highest body weight and those subjected to SER plus TS had the lowest body weight. Hot and cold carcass weights were influenced by Diet (*P* < 0.01; Table 2) but not by ST or Diet × ST interaction. Both hot and cold carcass weights were high in the BG-DW group, low in SER groups, and intermediate in BG group. However, carcass yield was not influenced by Diet, ST, or Diet × ST.

**Table 2:** Effects of diet and transportation stress on live body weights and carcass weights in goats.

Item	ST	Diet			n	SEM	*P*–value by ANOVA		
		SER	BG	BG-DW			Diet	ST	Diet × ST
**Live weights, kg**									
Day before ST	TS	60.0	68.5	68.3	6	3.43	0.076	–	–
	NTS	65.7	67.8	73.3	6				
Immediately before ST	TS	58.3	67.0	66.7	6	3.28	0.053	–	–
	NTS	63.5	66.2	71.5	6				
After ST	TS	57.3^b^	66.3^a^	66.2^a^	6	3.23	0.041	0.264	0.590
	NTS	62.7^b^	65.5^ab^	70.7^a^	6				
**Carcass weights, kg**									
Hot carcass	TS	23.7^b^	26.2^ab^	27.8^a^	6	1.53	0.013	0.404	0.902
	NTS	24.2^b^	27.0^ab^	29.7^a^	6				
Cold carcass	TS	21.2^b^	24.2^ab^	26.4^a^	6	1.56	0.008	0.413	0.939
	NTS	22.5^b^	24.6^ab^	27.8^a^	6				
**Carcass yield, %**									
Carcass yield	TS	41.6	39.4	42.1	6	1.20	0.197	0.657	0.138
	NTS	38.4	41.3	42.0	6				

*SER* Serala sericea hay-fed, *BG* bermudagrass hay-fed, *BG-DW* bermudagrass hay-fed dewormed, *ST* Stress Treatment group (*NTS* non-transported, *TS* transported).

^ab^Means within a row with different superscripts differ significantly (*P* < 0.05) by LSD test.

### *Longissimus* muscle composition

Crude protein main effect means were higher (*P* < 0.05) in SER (22.5 ± 0.22) and BG-DW (22.3 ± 0.22) groups compared to the BG group (21.6 ± 0.22); however, fat, crude fiber, and ash percentages were not influenced by ST or Diet (Table 3).

**Table 3:** Effects of diet and transportation stress on proximate composition (%) of meat (L*ongissimus dorsi*) in goats.

Item	ST	Diet			n	SEM	*P*–value by ANOVA		
		SER	BG	BG-DW			Diet	ST	Diet × ST
Moisture	TS	75.03	75.81	75.33	6	0.617	0.876	0.604	0.266
	NTS	75.98	74.89	76.09	6				
Ash	TS	1.08	1.42	1.04	6	0.044	0.494	0.676	0.417
	NTS	1.09	1.11	1.11	6				
Protein	TS	22.6^a^	21.26^b^	22.98^a^	6	0.316	0.016	0.249	0.014
	NTS	22.42	21.88	21.63	6				
Crude fat	TS	2.65	2.69	2.81	6	0.081	0.792	0.669	0.027
	NTS	2.87	2.76	2.60	6				

^ab^Means within a row with different superscripts differ significantly (*P* < 0.05) by LSD test. *SER* Serala sericea hay-fed, *BG* bermudagrass hay-fed, *BG-DW* bermudagrass hay-fed dewormed, *ST* Stress Treatment (*NTS* non-transported, *TS* transported).

### Plasma glucose, creatine, and creatinine concentrations

**Figure 1:**
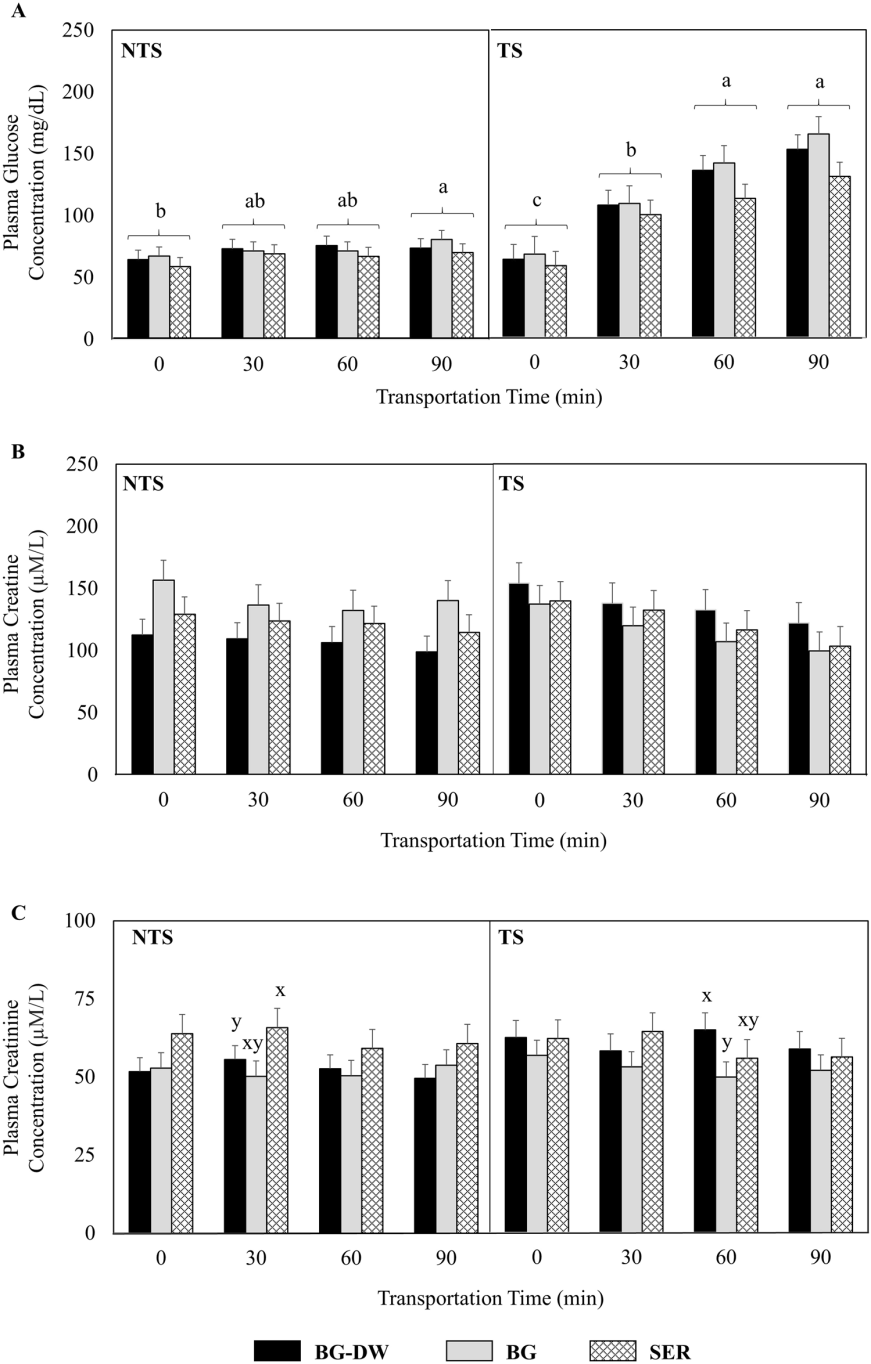
Effects of Diet (*SER* sericea hay-fed, *BG* bermudagrass hay-fed, *BG-DW* bermudagrass hay-fed dewormed) and stress treatment (ST; *NTS* non-transported, *TS* transported) on plasma (**A**) glucose (Diet, *P* < 0.05; Time, *P* < 0.01; ST × Time, *P* < 0.01), (**B**) creatine (Time, *P* < 0.01), and (**C**) creatinine (Diet, *P* < 0.01) concentrations in goats. ^abc^Within a ST group, Time main effect means with different letters differ significantly (*P* < 0.05) by LSD test. ^xy^Diet means within a cluster with different letters differ significantly (*P* < 0.05) by LSD test.

Plasma glucose concentrations were lower (*P* < 0.05) in SER diet group than in BG-DW or BG groups. There were significant Time (*P* < 0.01) and ST × Time interaction (*P* < 0.01) effects on glucose concentrations, since the levels in the TS group increased steadily over transportation time, while such a pattern was absent in the NTS group (Fig. 1A). Plasma creatine levels were not influenced by any interaction effects studied other than ST × Time. Overall mean creatine concentrations decreased over transportation time (Time, *P* < 0.01) in the TS group but not in the NTS group (Fig. 1B). Plasma creatinine levels were not influenced by any of the factors studied other than Diet effect. Plasma creatinine concentrations were high in SER diet group, low in BG, and intermediate in the BG-DW group (*P* < 0.01; Fig. 1C).

### Plasma metabolomics

**Figure 2:**
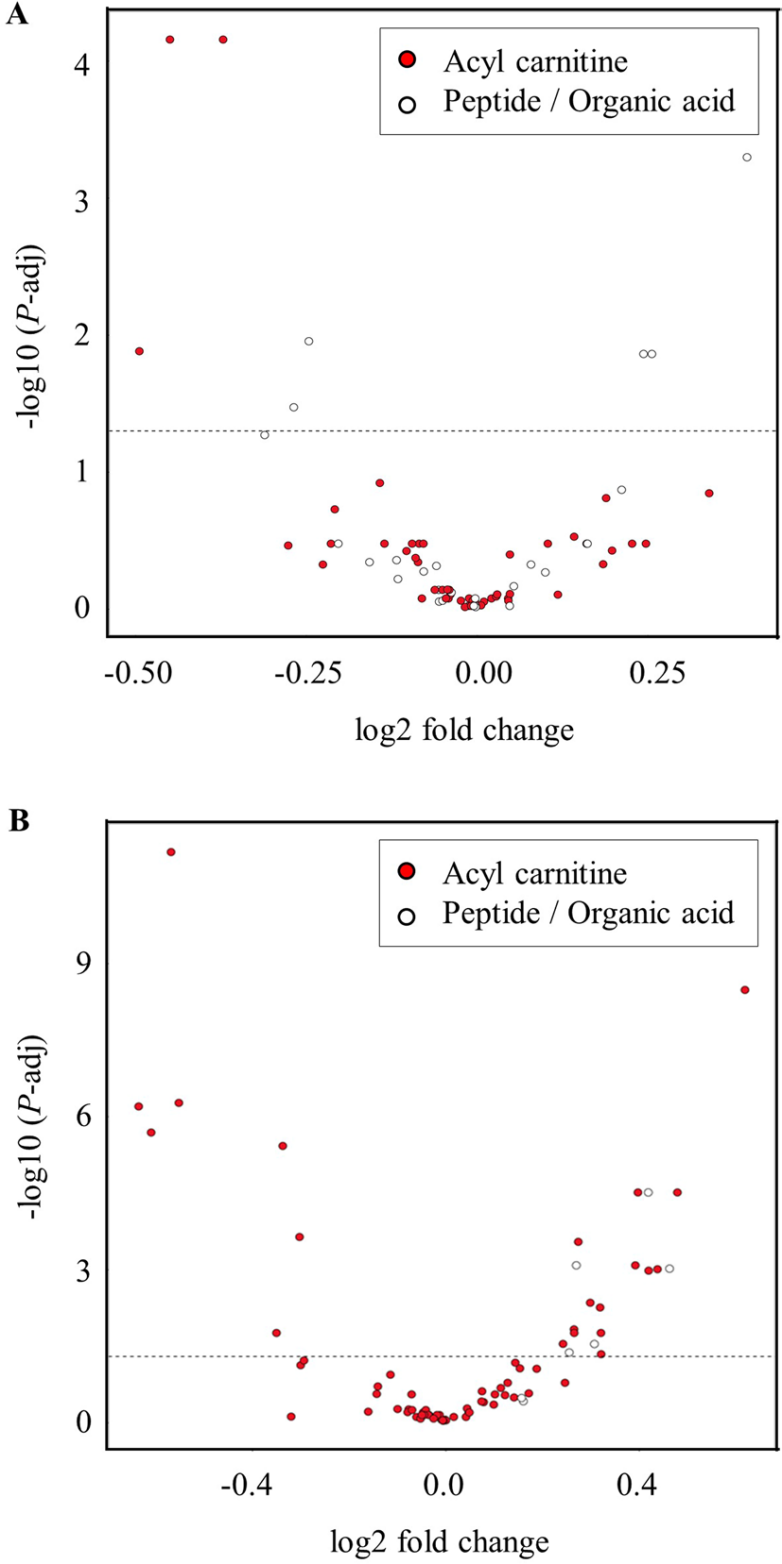
Volcano plot showing metabolites (acylcarnitines and peptides) significantly affected by Diet when (**A**) SER (sericea hay-fed) group was compared with BG (Bermudagrass hay-fed) group and when (**B**) SER group was compared with BG-DW (bermudagrass-fed dewormed goats) group across stress treatments in goats.

After conducting a preliminary PCA to study the clustering of all three dietary treatments for all plasma metabolites, specific comparisons of interest were carried out to identify the metabolites that were significantly affected by the factors studied. The relative abundance of all metabolites when SER and BG diets were compared was visualized by means of a heat map (see Supplementary Fig. S1). A volacano plot of acylcarnitines and peptides comparing SER and BG diets (Fig. 2A) revealed that SER diet group had significantly lower (*P* < 0.05) trans-hydroxyproline, carnosine, and aspartic acid, and higher (*P* < 0.05) butyryl carnitine, valeryl carnitine, alpha aminoadipic acid, butyric acid, and ornithine concentrations. The concentrations of these metabolites in the two diet groups and the related biological pathways they are involved in are shown in Table 4. The relative abundance of all metabolites when SER and BG-DW diets were compared was visualized by means of a heat map (see Supplementary Fig. S1). A volcano plot of acyl carnitines and peptides/organic acids comparing SER and BG-DW diets (Fig. 2B) revealed that Diet had effects (*P* < 0.05) on 21 acyl carnitines and 5 peptides/organic acids. In addition to higher butyryl carnitine, valeryl carnitine, alpha aminoadipic acid, and ornithine concentrations, carnitine, citrulline, and hydroxyvaleryl carnitine concentrations were also higher (*P* < 0.01) in the SER group when compared with BG-DW group (Table 5).

**Table 4:** Metabolites that were significantly affected in SER versus BG diet comparison (n = 12 goats/diet group). *SER* Serala sericea hay-fed, *BG* Bermudagrass hay-fed.

Metabolite	Diet (mean)		SEM	Fold change	Log2 fold change	Percent difference	*P-*value	Related pathway
	SER	BG						
Butyryl carnitine	0.13	0.09	0.04	0.73	− 0.45	− 30.8	< 0.0001	β-Oxidation of fatty acids
Valeryl carnitine	0.10	0.08	0.04	0.77	− 0.37	− 20.0	< 0.0001	β-Oxidation of fatty acids
Trans-Hydroxyproline	16.76	22.04	0.05	1.32	0.40	120.0	0.0005	Metabolism of collagen
Alpha-aminoadipic acid	0.73	0.61	0.04	0.84	− 0.25	− 16.4	0.0111	Degradation of lysine
Butyric acid	2.96	2.10	0.07	0.71	− 0.49	− 29.1	0.0131	Production of energy, anti-inflammatory
Carnosine	13.84	16.53	0.05	1.19	0.26	19.4	0.0137	Metabolism of histidine
Aspartic acid	4.94	5.85	0.04	1.18	0.24	18.4	0.0137	Urea cycle, recycling of ammonia
Ornithine	43.57	36.17	0.05	0.83	− 0.27	− 16.9	0.0336	Urea cycle, metabolism of citrulline

**Table 5:** Metabolites that were significantly affected in SER versus BG-DW diet comparison (n = 12 goats/diet group). *SER* Serala sericea hay-fed, *BG-DW* bermudagrass hay-fed dewormed.

Metabolite	Diet (mean)		SEM	Fold change	Log2 fold change	Percent difference	*P*-value	Related pathway(s)
	SER	BG-DW						
Valeryl carnitine	0.10	0.07	0.03	0.67	− 0.57	− 30.0	< 0.0001	β-Oxidation of fatty acids
Trans-hydroxyproline	16.76	25.74	0.04	1.54	0.62	53.6	< 0.0001	Metabolism of collagen
Butyryl carnitine	0.13	0.09	0.05	0.68	− 0.55	− 30.8	< 0.0001	β-Oxidation of fatty acids
Carnitine	11.34	7.30	0.05	0.64	− 0.64	− 35.6	< 0.0001	β-Oxidation of fatty acids
Citrulline	143.04	93.75	0.04	0.66	− 0.61	− 34.5	< 0.0001	Metabolism of Arg, immunity
Hydroxyvaleryl carnitine	0.05	0.04	0.03	0.79	− 0.34	− 20.0	< 0.0001	β-Oxidation of fatty acids
Alpha-ketoglutaric	30.44	40.69	0.04	1.34	0.42	33.7	< 0.0001	TCA cycle
Methionine	12.26	16.15	0.04	1.32	0.40	31.7	< 0.0001	Metabolism of betaine, Gly, Ser
Octadecanoyl carnitine	0.07	0.09	0.05	1.39	0.48	28.6	< 0.0001	β-Oxidation of fatty acids
Alpha-aminoadipic acid	0.73	0.59	0.04	0.81	− 0.30	− 19.1	< 0.0002	Degradation of lysine
Histidine	30.23	36.54	0.04	1.21	0.27	20.9	< 0.0003	Metabolism of β-alanine
Hydroxyhexadecenoyl carnitine	0.02	0.02	0.05	1.31	0.39	0	< 0.0008	β-Oxidation of fatty acids
Succinic acid	2.86	3.45	0.03	1.21	0.27	20.6	< 0.0008	TCA cycle, ETC
Taurine	23.16	31.91	0.07	1.38	0.46	37.8	< 0.0009	Biosynthesis of bile acids
Hexadecenoyl carnitine	0.05	0.07	0.06	1.35	0.44	40.0	< 0.0010	β-Oxidation of fatty acids
Hydroxyoctadecenoyl carnitine	0.01	0.01	0.05	1.34	0.42	0	0.0010	β-Oxidation of fatty acids
Threonine	37.51	46.14	0.05	1.23	0.30	23.0	0.0044	Metabolism of glycine, serine
Aspartic acid	4.94	6.16	0.05	1.25	0.32	24.7	0.0055	Urea cycle, recycling of NH_3_
Hexadecenoyl carnitine	0.03	0.03	0.04	1.20	0.27	0	0.0147	β-Oxidation of fatty acids
Octadecenoyl carnitine	0.05	0.06	0.06	1.25	0.32	20.0	0.0173	β-Oxidation of fatty acids
Serine	36.96	28.99	0.06	0.78	− 0.35	− 21.6	0.0173	Metabolism of Gly, Met
Tyrosine	35.80	43.02	0.05	1.20	0.27	20.2	0.0173	Synthesis of catecholamines
Hydroxyhexadecadienoylcarnitine	0.01	0.01	0.05	1.18	0.24	0	0.0283	β-Oxidation of fatty acids
β-Hydroxybutyric acid	339.77	420.40	0.06	1.24	0.31	23.7	0.0285	Fatty acid metabolism
Pyruvic acid	38.32	45.73	0.05	1.19	0.26	19.3	0.0417	TCA cycle, gluconeogenesis
Tetradecanoyl carnitine	0.02	0.02	0.06	1.25	0.32	0	0.0451	β-Oxidation of fatty acids

**Figure 3:**
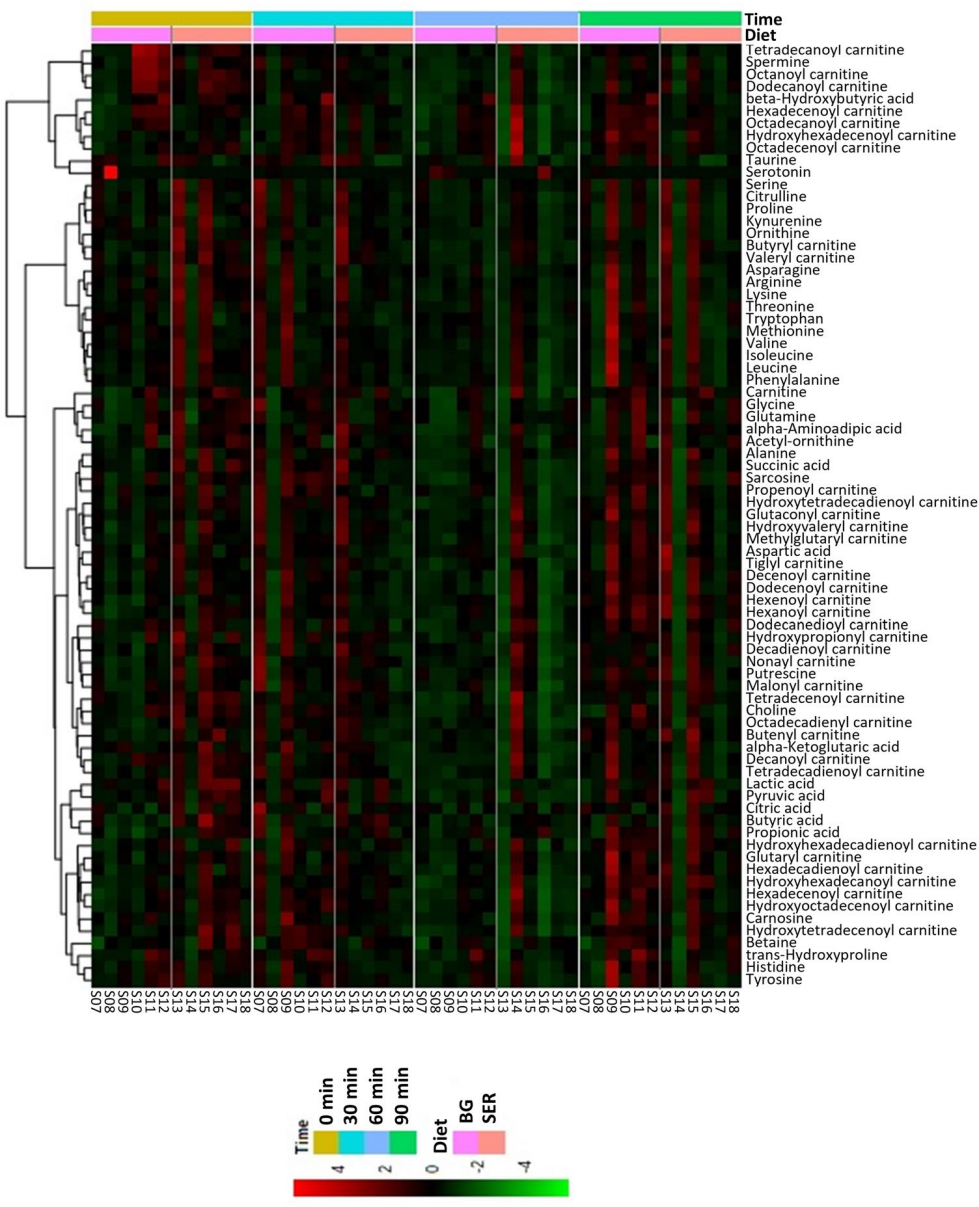
Hierarchical clustering heat map of plasma metabolites in goats in response to Diet and sampling time when SER (sericea hay-fed) was compared with BG (bermudagrass hay-fed) within the NTS (non-transported) group.

**Figure 4:**
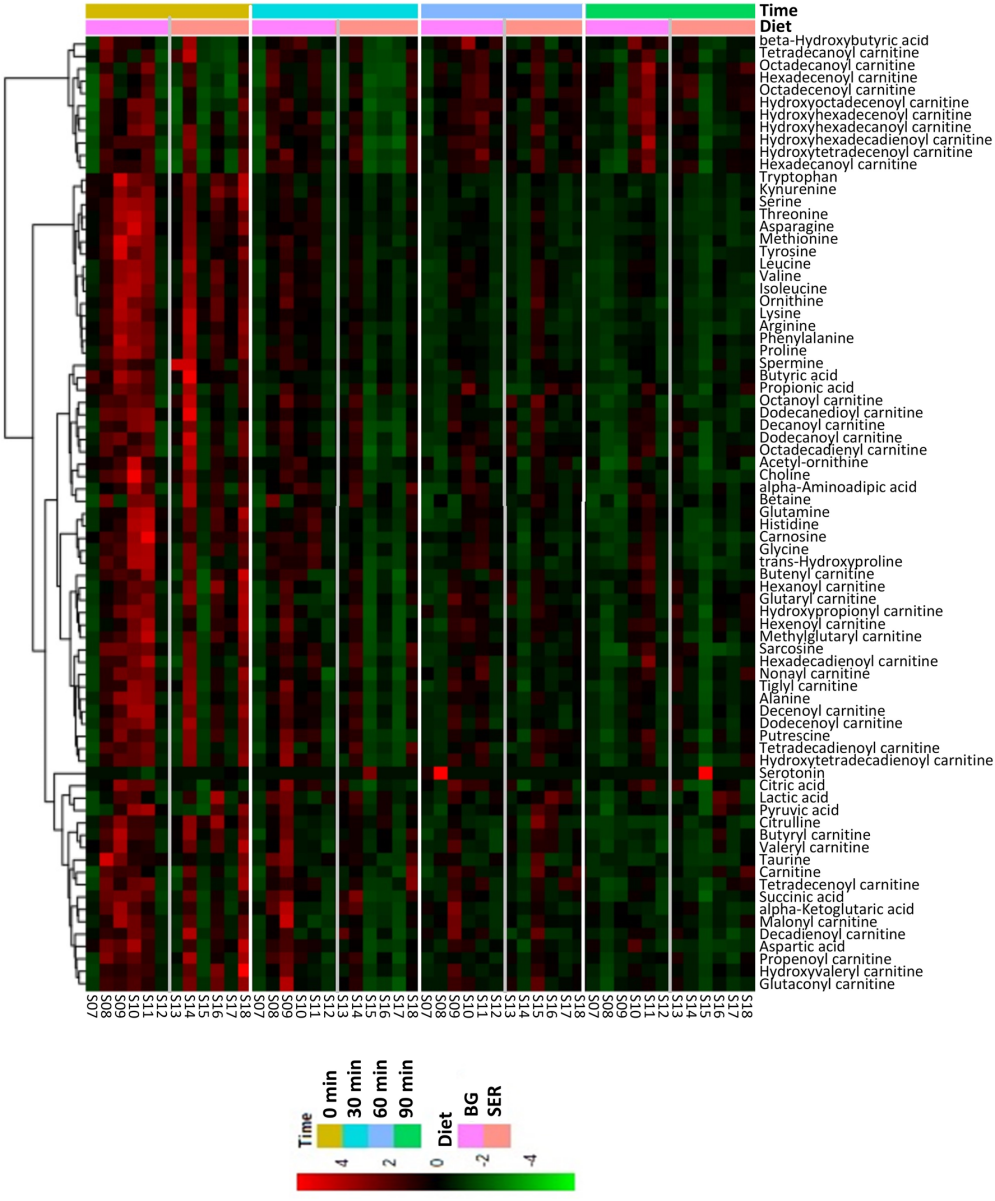
Hierarchical clustering heat map of plasma metabolites in goats in response to Diet and transportation time when SER (sericea hay-fed) was compared with BG (bermudagrass hay-fed) within the TS (transported) group.

**Figure 5:**
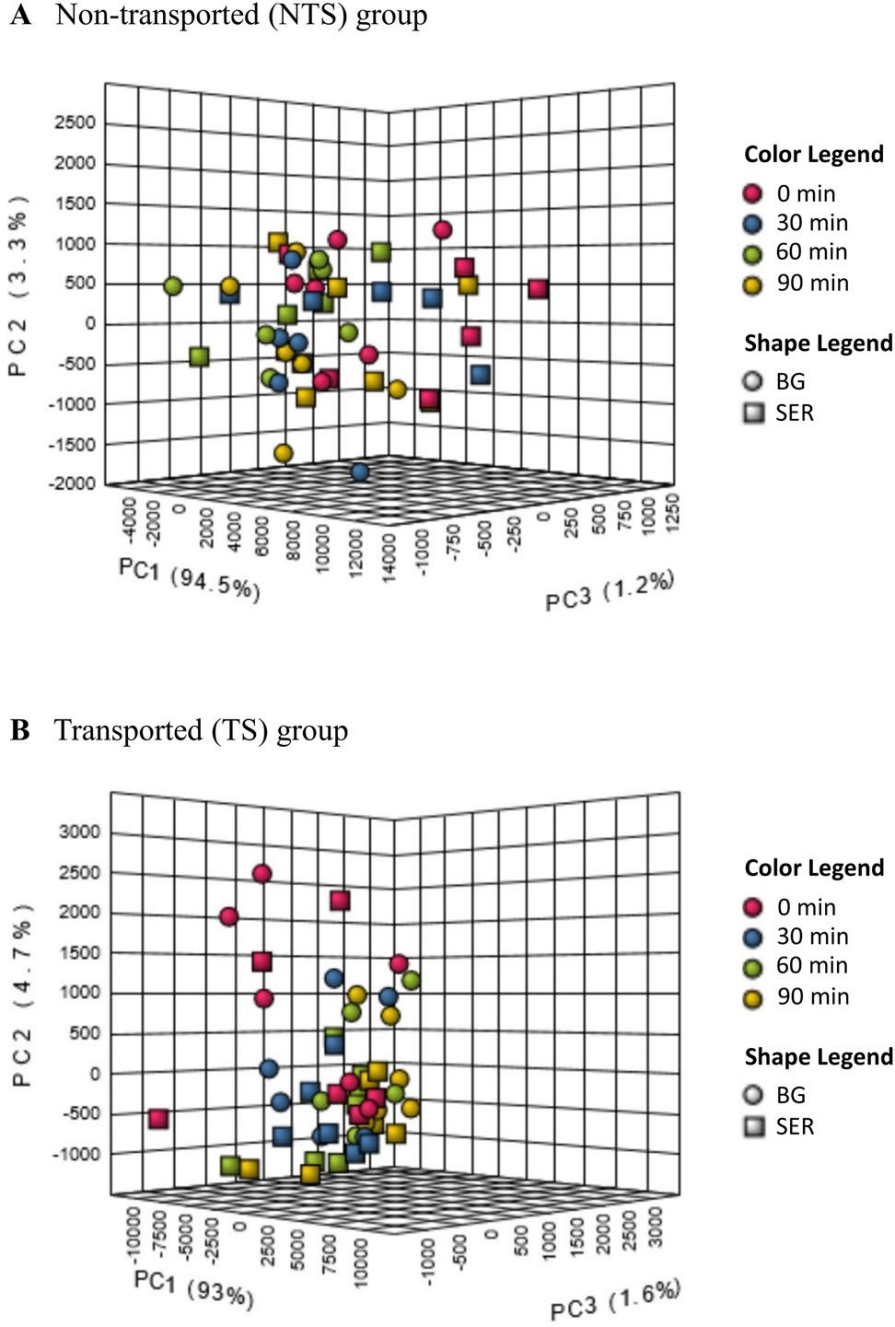
PCA plot showing the effects of Diet and sampling time on metabolites when SER (sericea hay-fed) group was compared with BG (bermudagrass hay-fed) group in (**A**) NTS (non-transported) and (**B**) TS (transported) groups of goats.

To evaluate changes in plasma metabolome caused by Diet (SER vs. BG) effect within an ST group, a hierarchical clustering analysis using the degree of abundance was conducted in which metabolites with identical abundance patterns were clustered together. Clustering was visualized by means of heatmap dendograms shown in Figs. 3 and 4 for NTS and TS groups, respectively. Repeated measures analysis of data separated by ST, and PCA (Fig. 5) plot revealed that a total of only 10 compounds were influenced (*P* < 0.05) by Diet when SER and BG groups were compared in the NTS group (Fig. 5A). However, in the TS group, 25 amino acids, 14 acylcarnitines, and 7 other organic acids and compounds were significantly (*P* < 0.05) affected by Diet (Fig. 5B).

**Figure 6:**
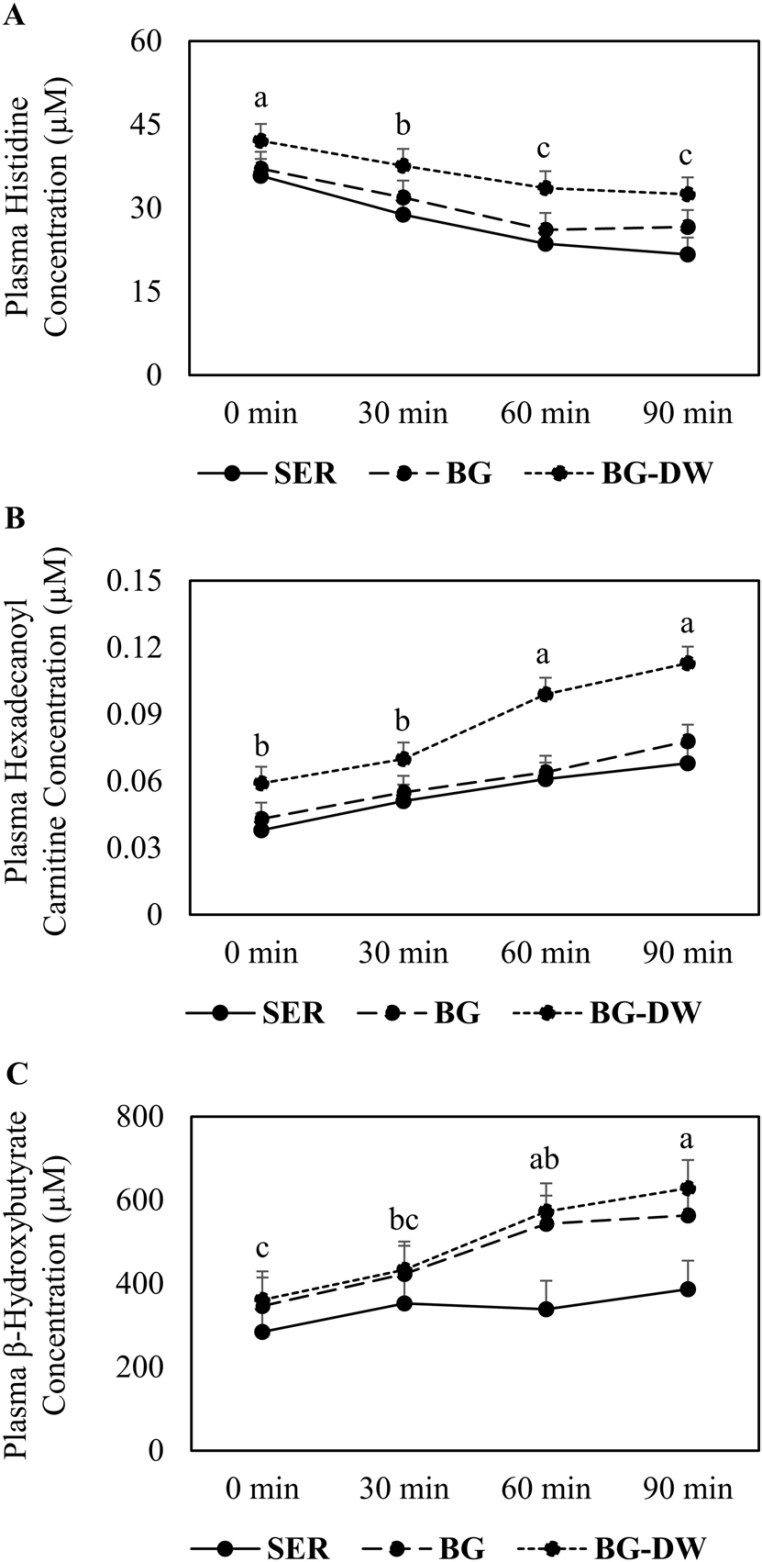
Changes in the concentrations of (**A**) histidine, (**B**) hexadecanoyl carnitine, and (**C**) β-hydroxybutyrate as affected by transportation time (0 min, 30 min, 60 min, 90 min) in goats in each Diet (*SER* sericea-hay fed, *BG* bermudagrass hay-fed, *BG-DW* bermudagrass hay-fed dewormed) group. Error bars represent SEM. ^abc^Main effect Time means with different letters differ significantly by LSD (*P* < 0.05) test.

Notable among the three metabolites influenced by Diet in the NTS group was the amino acid lysine concentration, which was higher (*P* < 0.01) in the SER group compared to BG and BG-DW groups (Table 6). In the TS group, however, 10 amino acids (Table 7) and 15 acylcarnitines and other organic compounds (Table 8) were affected (*P* < 0.05) by Diet. The SER group had the lowest glycine, alanine, threonine, taurine, trans-hydroxyproline, methionine, and histidine concentrations and highest citrulline concentrations among the three diet treatments (Table 7). Carnitine, butyryl carnitine, and valeryl carnitine concentrations were higher (*P* < 0.05) in the SER groups compared to BG or BG-DW groups; however, 8 other acylcarnitines were highest in the BG-DW group (Table 8). Transportation time had an effect (*P* < 0.05) on 21 amino acids and 18 acyl carnitines and other organic acids. For a majority of amino acids, the concentrations decreased over Time in all three dietary treatments and for a majority of the acyl carnitines, the concentrations increased over Time. An example of a compound in each metabolite group that changed over time is shown in Fig. 6. Diet × Time interaction effect was not significant for any of the metabolites.

**Table 6:** Effects of dietary treatment (Diet; n = 12 goats/diet group) and sampling time (Time) on plasma metabolite concentrations (µM) in goats not subjected to transportation stress. ^ab^Diet main effect means within a row with different superscripts differ significantly (*P* < 0.05) by LSD test. *SER* Serala sericea hay-fed, *BG* bermudagrass hay-fed, *BG-DW* bermudagrass hay-fed dewormed.

Item	Diet			SEM	*P*-value by ANOVA		
	SER	BG	BG-DW		Diet	Time	Diet × Time
Acetyl-ornithine	7.4^a^	5.7^b^	7.8^a^	0.45	0.004	0.395	0.999
Ornithine	45.3^a^	35.5^b^	41.5^ab^	2.22	0.012	0.105	0.993
Lysine	56.3^a^	47.9^b^	41.5^b^	2.66	0.001	0.024	0.985
Choline	7.5	7.0	7.2	0.36	0.619	0.002	0.993
Lactic acid	2526.3	2046.4	2697.1	266.62	0.177	0.002	0.901
Hexanoyl carnitine	0.067	0.064	0.065	0.0023	0.538	< 0.001	0.669
Hexenoyl carnitine	0.031	0.032	0.032	0.0013	0.842	< 0.001	0.867
Octanoyl carnitine	0.019	0.019	0.016	0.0016	0.403	< 0.001	0.151
Dodecanoyl carnitine	0.026	0.027	0.026	0.0017	0.869	0.001	0.325
Tetradecanoyl carnitine	0.008	0.007	0.007	0.0003	0.378	0.421	0.417

**Table 7:** Effects of dietary treatment (Diet; n = 12 goats/diet group) and transportation time (Time) on concentrations (µM) of plasma amino acids and intermediates in goats subjected to transportation stress for 90 min. ^abc^Diet main effect means within a row with different superscripts differ significantly (*P* < 0.05) by LSD test. *SER* Serala sericea hay-fed, *BG* bermudagrass hay-fed, *BG-DW* bermudagrass hay-fed dewormed.

Item	Diet			SEM	*P*-value by ANOVA		
	SER	BG	BG-DW		Diet	Time	Diet × Time
Glycine	413.8^b^	510.7^a^	475.9^ab^	23.97	0.017	0.004	1.000
Alanine	152.0^b^	162.7^ab^	168.2^a^	4.41	0.033	0.072	0.587
Serine	29.5^ab^	32.6^a^	26.7^b^	2.51	0.016	< 0.001	0.870
Proline	63.4	62.9	65.3	2.91	0.644	< 0.001	0.788
Valine	171.5	168.6	172.1	8.95	0.945	< 0.001	0.985
Threonine	30.9^b^	35.3^b^	44.4^a^	3.01	< 0.001	< 0.001	0.989
Taurine	20.9^b^	24.5^b^	36.4^a^	2.60	< 0.001	0.011	0.897
Trans-Hydroxyproline	14.1^b^	21.7^a^	24.3^a^	1.06	< 0.001	0.252	0.995
Leucine	101.7	96.3	105.6	5.50	0.472	0.002	0.982
Isoleucine	62.3	64.4	62.9	3.36	0.881	< 0.001	0.992
Asparagine	16.0	18.3	18.7	1.87	0.104	< 0.001	0.960
Glutamine	102.4	108.6	103.3	9.93	0.886	0.002	0.994
Methionine	10.5^c^	12.6^b^	14.4^a^	0.72	< 0.001	< 0.001	0.722
Histidine	27.5^b^	30.4^b^	36.5^a^	1.65	< 0.001	< 0.001	0.991
alpha-Aminoadipic acid	0.7	0.6	0.6	0.03	0.469	0.154	0.881
Phenylalanine	32.2	30.1	33.1	1.45	0.117	< 0.001	0.699
Arginine	105.3	101.7	96.7	6.43	0.338	< 0.001	0.896
Acetyl-ornithine	6.9	7.4	7.1	0.45	0.765	0.269	0.999
Citrulline	156.8^a^	112.1^b^	89.8^b^	8.95	< 0.001	< 0.001	0.976
Tyrosine	33.3	35.9	37.8	2.57	0.322	< 0.001	0.855
Tryptophan	14.9	14.6	14.9	1.28	0.925	< 0.001	0.886
Kynurenine	2.5	2.4	2.5	0.25	0.769	< 0.001	0.664
Carnosine	12.3^b^	17.3^a^	13.2^b^	0.85	< 0.001	< 0.001	0.910
Ornithine	41.8	36.9	37.4	2.5	0.089	< 0.001	0.936
Lysine	41.5	37.0	38.3	3.77	0.368	< 0.001	0.768

**Table 8:** Effects of dietary treatment (Diet; n = 12 goats/diet group) and transportation time (Time) on plasma carnitine and organic acid concentrations (µM) in goats subjected to transportation stress for 90 min. ^abc^Diet main effect means within a row with different superscripts differ significantly (*P* < 0.05) by LSD test. *SER* Serala sericea hay-fed, *BG* bermudagrass hay-fed, *BG-DW* bermudagrass hay-fed dewormed.

Item	Diet			SEM	*P*-value by ANOVA		
	SER	BG	BG-DW		Diet	Time	Diet × Time
Carnitine	21.55^a^	17.77^b^	12.82^c^	1.3237	< 0.001	0.004	0.997
Butyryl carnitine	0.115^a^	0.091^b^	0.101^ab^	0.0068	0.049	0.004	0.994
Valeryl carnitine	0.094^a^	0.078^b^	0.067^b^	0.0049	0.001	0.007	0.909
Hexanoyl carnitine	0.068	0.064	0.064	0.0021	0.188	0.001	0.305
Nonayl carnitine	0.018	0.019	0.019	0.0007	0.648	0.004	0.273
Hydroxytetradecenoyl carnitine	0.015^b^	0.015^b^	0.018^a^	0.0008	< 0.001	< 0.001	0.561
Hexadecenoyl carnitine	0.031^b^	0.030^b^	0.041^a^	0.0017	< 0.001	< 0.001	0.304
Hexadecanoyl carnitine	0.055^b^	0.060^b^	0.085^a^	0.0047	< 0.001	< 0.001	0.517
Hydroxyhexadecadienoyl carnitine	0.008^b^	0.008^b^	0.010^a^	0.0005	0.001	< 0.001	0.523
Hydroxyhexadecenoyl carnitine	0.018^c^	0.021^b^	0.024^a^	0.0013	< 0.001	< 0.001	0.115
Hydroxyhexadecanoyl carnitine	0.010^b^	0.011^b^	0.013^a^	0.0007	0.060	0.001	0.190
Octadecenoyl carnitine	0.052^b^	0.053^b^	0.071^a^	0.0040	< 0.001	< 0.001	0.269
Octadecanoyl carnitine	0.065^b^	0.074^b^	0.092^a^	0.0057	< 0.001	< 0.001	0.812
Hydroxyoctadecenoyl carnitine	0.010^b^	0.012^b^	0.016^a^	0.0009	< 0.001	< 0.001	0.105
Betaine	110.4^b^	102.9^b^	144.7a	10.24	0.027	0.791	0.999
Choline	6.17	6.47	6.37	0.383	0.757	< 0.001	0.866
beta-Hydroxybutyric acid	340.5^b^	468.9^a^	498.7^a^	35.48	0.004	0.004	0.793
Citric acid	124.0^b^	175.3^a^	167.8^a^	10.32	0.005	0.558	0.999
Butyric acid	2.13	1.95	2.63	0.318	0.316	0.004	0.999
Succinic acid	2.71^b^	2.85^b^	3.29^a^	0.117	0.001	0.003	0.740
Propionic acid	2.49	2.57	2.76	0.190	0.626	0.901	0.909

## Discussion

Meat goat management practices that increase animal stress may alter the normal course of conversion of muscle to meat, leading to inferior meat quality^3^. Stress in animals can negatively affect animal weight gain, immune function, and physiological status that in turn can lead to poorer health and lower carcass yield and economic returns^21^. Diets high in CT, such as SL in the form of hay or pellets, or SL feed as diet supplement have shown to ameliorate the negative effects of stress in small ruminants, possibly due to the high polyphenol content^22^.

Animal management during the 24-h period prior to slaughter is crucial not only for animal welfare reasons, but also for profitability. Diet had a significant effect when body weights were measured after ST when the gut contents would have been partially evacuated. Feed was withdrawn from these goats the previous evening and transportation stress is likely to hasten gut emptying as defecation is a frequently noticed stress response in ruminants. Stress combined with light physical exercise can accelerate gastrointestinal tract emptying, while vigorous exercise slows gastric emptying^23^. The lowest body weight in the SER group, particularly when exposed to transportation stress, among the three dietary treatment groups shows that the Serala diet (SER) is likely inferior to Bermudagrass diet in nutritional quality. In fact, body weights measured the day before ST (final weight) was also lower for SER than BG fed goats. Additionally, there is evidence that absence of gastrointestinal parasites in goats improved body weight gain, since the goats subjected to BG-DW plus NTS had the highest body weight among the three dietary treatments. However, Moore et al.^24^ reported that SL hay feeding, regardless of gastrointestinal parasite load, improved performance, including average daily gain, in 6-month-old Spanish × Kiko goats relative to similar animals on Bermuda-grass based diets. In the current study, both hot and cold carcass weights were the highest in the BG-DW group, lowest in SER group, and intermediate in BG group. The diet effect on carcass weights was similar to that on live body weights measured after transportation. Mechineni^25^ reported that SL forage improved average daily gain and carcass yield in goats. The discrepancies between these studies and the present study can be attributed to the difference in the varieties of SL used, form of diet (fresh forage vs. hay), or both. For this study, we used ‘Serala’ hay while the other studies used AU grazer variety.

Creatine levels have been used to assess muscle protein mass in small ruminants^26^ and is considered to have protective action against oxidative stress^27^. The decrease in creatine concentration in blood over Time may indicate increased energy depletion, as well as susceptibility to oxidative stress due to transportation. Creatine metabolism results in the production of creatinine. Plasma creatinine concentrations were high in SER diet group, low in BG, and intermediate in the BG-DW group.

Blood metabolites, such as glucose and creatinine, have been used by researchers to monitor nutrient status and muscle mass^28^. Plasma glucose concentrations were lower and creatinine concentrations higher in the SER diet group than in BG-DW or BG groups in our study. These results are consistent with the findings of Turner et al.^28^, who also reported that circulating glucose concentrations were lower and creatinine concentrations higher in SL-fed bucklings of three different breed groups. It is likely that CT interfered with fiber digestion, leading to lower VFA production. Lower propionate production leads to low gluconeogenesis precursor and lower glucose production. Lower glucose combined with ST could have triggered higher cortisol release, leading to protein breakdown, for gluconeogenesis to overcome the deficit due to diet (SER). Lespedeza has been reported to be a forage of poorer quality compared to alfalfa^28^.

Crude protein percentages were higher in SER and BG-DW groups compared to the BG group. Lee et al.^29^ studied the effects of three different dietary regimens involving different hay-concentrate combinations on proximate composition of loin chops in Boer × Spanish kids and observed no changes in protein percentages. In the present study, the three diet treatments were comprised of equal proportions of roughage and concentrate. However, goats tend to consume a higher quantity of SER hay compared to BG hay that could result in greater protein content of *longissimus* muscle, although the effect may be more significant in adult goats than kids^30^. Barry et al.^31^ found that the crude protein percentage of *longissimus* muscle from tannin-fed lambs was higher compared with lambs fed the same diet supplemented with polyethylene glycol, a binding agent that negates the effects of CT.

Plasma metabolome can be influenced by genetic and environmental factors^13^. When SER group was compared individually with either BG or BG-DW for acylcarnitines, peptides and organic acids, there were significant effects of Diet on certain metabolites. Notable among the metabolites that were affected was butyric acid concentrations that were higher in the SER compared with the BG diet group. Butyric acid, a short-chain fatty acid resulting from rumen microbial fermentation of dietary fiber, has been shown to have anti-inflammatory properties^32^, and circulating levels of butyric acid have been reported to inversely correlate with inflammatory markers^33^. Butyrate has several beneficial effects in energy homeostasis, inflammation, and immune function in humans^34^. Higher butyric acid concentration in the SL-fed group may indicate better ability of goats to withstand oxidative stress. Koenig and Beauchemin^35^ reported that dietary condensed tannin supplementation increased rumen butyric acid concentrations in steers. Since butyrate has a greater potential for diffusional uptake^36^, a higher concentration of butyric acid in the rumen is likely to increase its circulating concentration.

Carnitine, butyryl carnitine, and valeryl carnitine concentrations were higher in the SER groups compared to BG or BG-DW groups; however, 8 other acylcarnitine concentrations were comparable to those in BG, but lower than in the BG-DW group. Carnitine is an important compound that has functions in intermediary metabolism. Carnitine plays a major role in importing acetyl-CoA into mitochondria and acylcarnitines function as a cofactor for the transport of long-chain fatty acids through the mitochondrial membrane^37^. In mitochondria, the acylcarnitine is reconverted to free carnitine and the respective long-chain acyl-CoA. The acyl-CoA is then used to produce ATP via β-oxidation and the TCA cycle^38^. Acylcarnitine may indicate the extent of β-oxidation of fatty acids and the energy status of animals^39^. Both medium- and long-chained acylcarnitines along with free carnitine have been identified in several plant species^40^. The higher concentrations of carnitine and two acylcarnitines in the SER group compared to the BG group suggests that goats could have obtained these metabolites through diet to a limited extent, in addition to being synthesized in the body. The SER group showed some level of fatty acid metabolism based on acylcarnitine concentrations, although not as high as the BG-DW group. The BG-DW group showed elevated concentrations of 8 other acylcarnitines and β-hydroxybutyrate concentration. Lower plasma β-hydroxybutyrate, a ketone body produced from lipolysis in the liver mitochondria, may indicate inhibition of β-oxidation of fatty acids^41^. It is likely that gastrointestinal parasite infection level influenced the extent of fatty acid metabolism in goats. Although carnitine and acylcarnitine concentrations were higher in the SER group compared to the BG group, the β-hydroxybutyrate concentration was lower in the SER group, indicating only a moderate level of fatty acid metabolism in this group.

Among the three metabolites influenced by Diet in the NTS group was the amino acid lysine concentration, which was higher in the SER group compared to BG and BG-DW groups. A diet deficient in lysine has been reported to decrease the whole-brain content of lysine and affect norepinephrine activity in the hypothalamus^42,43^. Lysine deficiency increases release of serotonin in the amygdala, with consequent changes in psychobehavioral responses to stress, such as increased stress-induced anxiety^44^, and also results in immunodeficiency^45^ in animal models. The higher plasma lysine concentration in the SER group indicates that CT-diet can confer better stress coping ability when they encounter typical preharvest livehaul situations. The metabolite α-aminoadipic acid, which was higher in the SER group compared to BG groups, is used by plants and animals to catabolize the amino acid lysine, while yeast and fungi use this derivative in the synthesis of lysine^46^. In a series of biochemical steps, lysine is catabolized to saccharopine, which is then converted to α-aminoadipic acid and then to acetyl CoA^47^. Lysine is also one of the precursors for the synthesis of carnitine^48^. Since lysine is one of the essential amino acids, its higher plasma concentration in SL-fed goats is likely from this leguminous plant.

In the TS group, the SER group had the lowest glycine, alanine, threonine, taurine, trans-hydroxyproline, methionine, and histidine concentrations and highest citrulline concentrations among the three diet treatments. The lower concentration of several amino acids in the SER group compared to the other groups studied does not mean that protein synthesis was impaired, since this correlates with intracellular amino acid pool rather than plasma amino acid concentrations^49^. Citrulline is an amino acid that plays an important role in the urea cycle. Arginine, which is synthesized from citrulline, has been reported to play an important role in modulating immune response during inflammatory stress in dairy cows^50^, and thus upregulated arginine-citrulline-nitric oxide pathway can help SER-fed goats cope up with oxidative stress and immune challenge^51^. Histidine and alanine are major amino acids involved in glucose metabolism in ruminant animals^52^. During times of feed deprivation or stress in dairy cows, gluconeogenesis is regulated by alanine to replenish glucose levels^52^. The lower alanine concentrations in the SER group compared to BG or BG-DW group indicates that carbohydrate metabolism was negatively affected by the high-CT diet. This point is further supported by lower concentrations of glucose and several other amino acids in the SER group, suggesting gluconeogenesis was negatively affected.

Transportation time had a significant effect on 21 amino acids and 18 acylcarnitines and other organic acids. With the exception of 3 amino acids, concentrations of all other amino acids decreased with Time in the TS goats, suggesting gluconeogenesis became inadequate to maintain energy levels with increasing stress. Stress due to transportation increases with transportation time in goats^3^. Feed was withdrawn from the goats in this experiment the previous evening; therefore, feed deprivation duration also increased with transportation time. In addition to depletion of amino acids, animals can also be susceptible to inflammatory reaction due to decrease in the concentration of histidine. Low plasma histidine concentrations are associated with inflammation^53^. Histidine supplementation has been shown to influence the concentrations of other metabolites in the blood, resulting in increased concentrations of several amino acids, including glycine and aspartic acid^54^. In contrast to down regulation of amino acid-related gluconeogenesis with increasing transportation time, long-chained acylcarnitine concentrations increased with increasing time in the TS group. Concentrations of free carnitine decreased and those of 9 acylcarnitines increased with time in the TS group, whereas only the concentrations of 2 acylcarnitines increased in the NTS group, suggesting that fatty acid metabolism is upregulated with increasing levels of stress in goats and is the predominant energy generating pathway during prolonged stressful situations. This is further supported by the increasing levels of β-hydroxybutyrate concentration with increasing time, although there was only a minimal increase in the SER group.

In conclusion, feeding Serala SL diet increased plasma concentrations of butyric acid, lysine, and citrulline, all of which can have beneficial effects in goats. Elevated butyric acid concentration can help with energy homeostasis, inflammation, and immune function, the amino acid lysine can be beneficial in reducing stress induced psychobehavioral changes and boost immune function, and citrulline can modulate immune response during inflammatory stress. There is evidence that fatty acid metabolism is the predominant energy production pathway in the SER group, although not at the level of dewormed, BG-fed goats. Several amino acid concentrations were lower in the SER group, suggesting that gluconeogenesis either did not sustain or happened only to a limited extent compared to the BG-DW group. The BG-DW goats were able to sustain energy production via elevated levels of both gluconeogenesis and β-oxidation of fatty acids as evidenced by higher amino acid and acylcarnitine concentrations. All three diet groups switched energy generation from gluconeogenesis to fatty acid metabolism when transportation time increased, as seen by decreasing amino acid concentrations and increasing medium- and long-chained acylcarnitines with increasing transportation time. Further studies are required to establish an ideal proportion of SL hay in the diet of meat goats, such that the beneficial effects are maximized, and the negative effects are minimized. Overall, SER diet can be beneficial to goats to some extent in enhancing stress coping abilities.

## Supplementary Information

Supplementary Figures.

## Data Availability

The datasets used during the current study are available from the corresponding author on reasonable request.
