# Supplementary material for: Plasma metabolomic profiles as affected by diet and stress in Spanish goats
Source: Sci Rep. 2021 Jun 15;11:12607. doi: 10.1038/s41598-021-91893-x (PMC8206094; doi:10.1038/s41598-021-91893-x)
Supplement: Supplementary file 1 — Supplementary Figures. [file 41598_2021_91893_MOESM1_ESM.docx]

**Plasma metabolomic profiles as affected by diet and stress in Spanish goats**

Phaneendra Batchu, Thomas H. Terrill, Brou Kouakou, Zaira M. Estrada-Reyes, Govind Kannan*

**Supplementary Information**


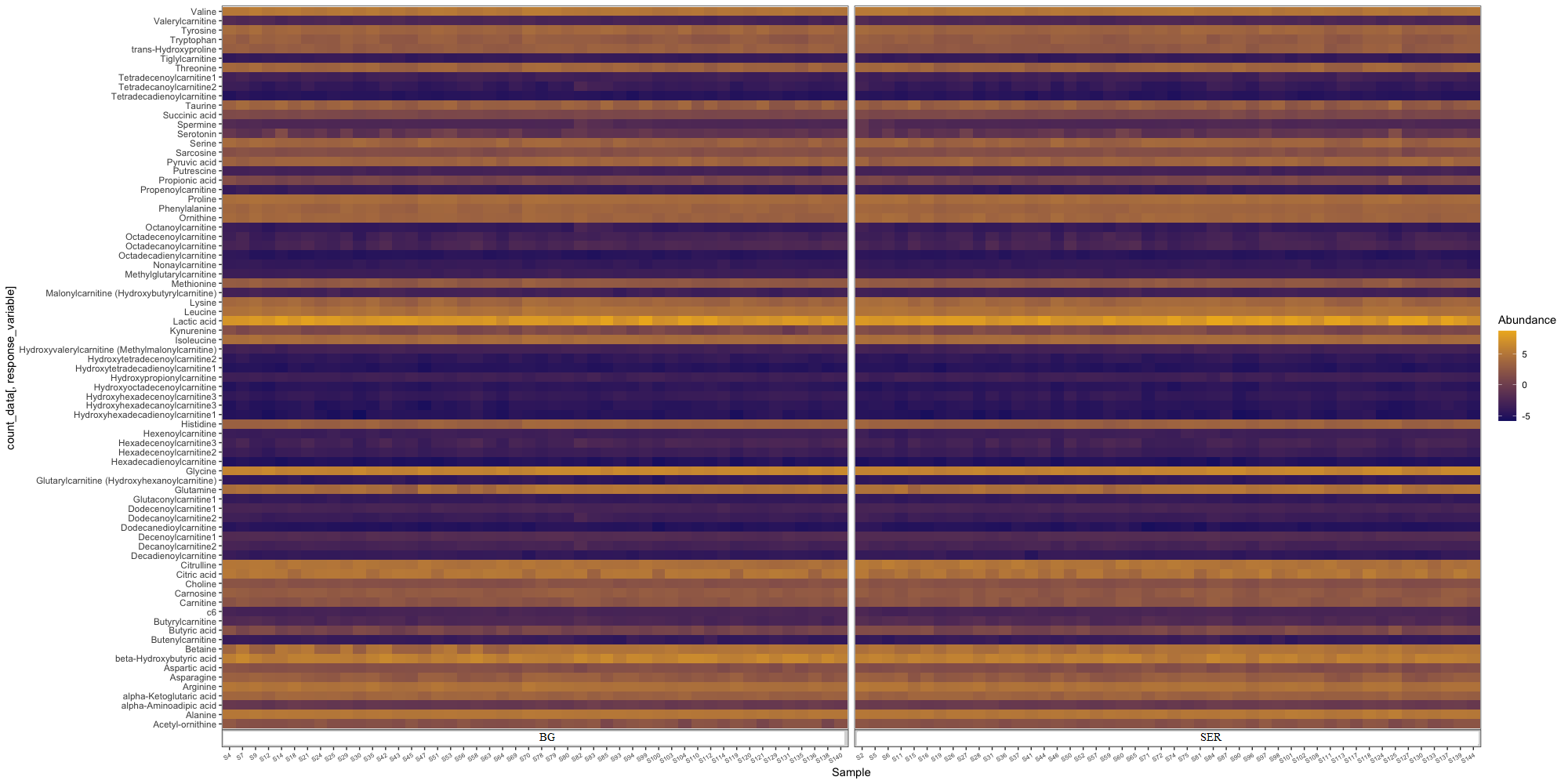


**Supplementary Fig. S1.** Heat map showing the relative abundance of metabolites when comparing Diet Treatments (BG: Bermudagrass hay-fed; SER: sericea hay-fed) in goats. The name of each metabolite is provided in the list to the left of the heat map.


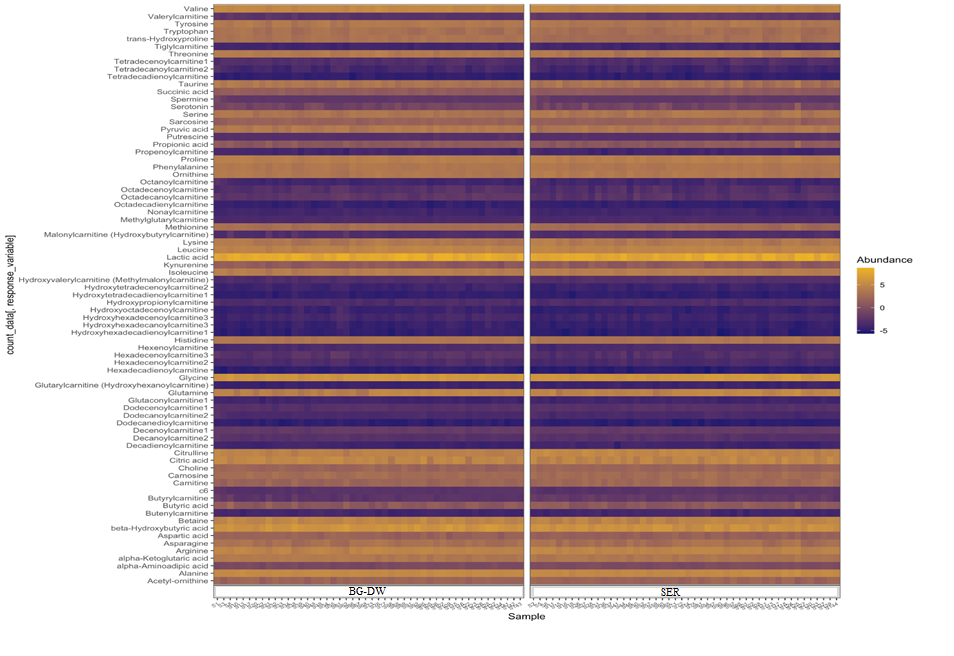


**Supplementary Fig. S2.** Heat map showing the relative abundance of metabolites when comparing Diet Treatments (BG-DW: Bermudagrass hay-fed dewormed; SER: sericea hay-fed) in goats. The name of each metabolite is provided in the list to the left of the heat map.
